# Neural microprobe modelling and microfabrication for improved implantation and mechanical failure mitigation

**DOI:** 10.1098/rsta.2021.0007

**Published:** 2022-07-25

**Authors:** Eve McGlynn, Finlay Walton, Rupam Das, Hadi Heidari

**Affiliations:** Microelectronics Lab (meLAB), James Watt School of Engineering, University of Glasgow, Glasgow G12 8QQ, UK

**Keywords:** brain implantable device, device modelling, polyimide, failure mitigation, flexible probe

## Abstract

Careful design and material selection are the most beneficial strategies to ensure successful implantation and mitigate the failure of a neural probe in the long term. In order to realize a fully flexible implantable system, the probe should be easily manipulated by neuroscientists, with the potential to bend up to 90°. This paper investigates the impact of material choice, probe geometry, and crucially, implantation angle on implantation success through finite-element method simulations in *COMSOL Multiphysics* followed by cleanroom microfabrication. The designs introduced in this paper were fabricated using two polyimides: (i) PI-2545 as a release layer and (ii) photodefinable HD-4110 as the probe substrate. Four different designs were microfabricated, and the implantation tests were compared between an agarose brain phantom and lamb brain samples. The probes were scanned in a 7 T PharmaScan MRI coil to investigate potential artefacts. From the simulation, a triangular base and 50 µm polymer thickness were identified as the optimum design, which produced a probe 57.7 µm thick when fabricated. The probes exhibit excellent flexibility, exemplified in three-point bending tests performed with a DAGE 4000Plus. Successful implantation is possible for a range of angles between 30° and 90°.

This article is part of the theme issue ‘Advanced neurotechnologies: translating innovation for health and well-being’.

## 1. Introduction

**Figure 1.**
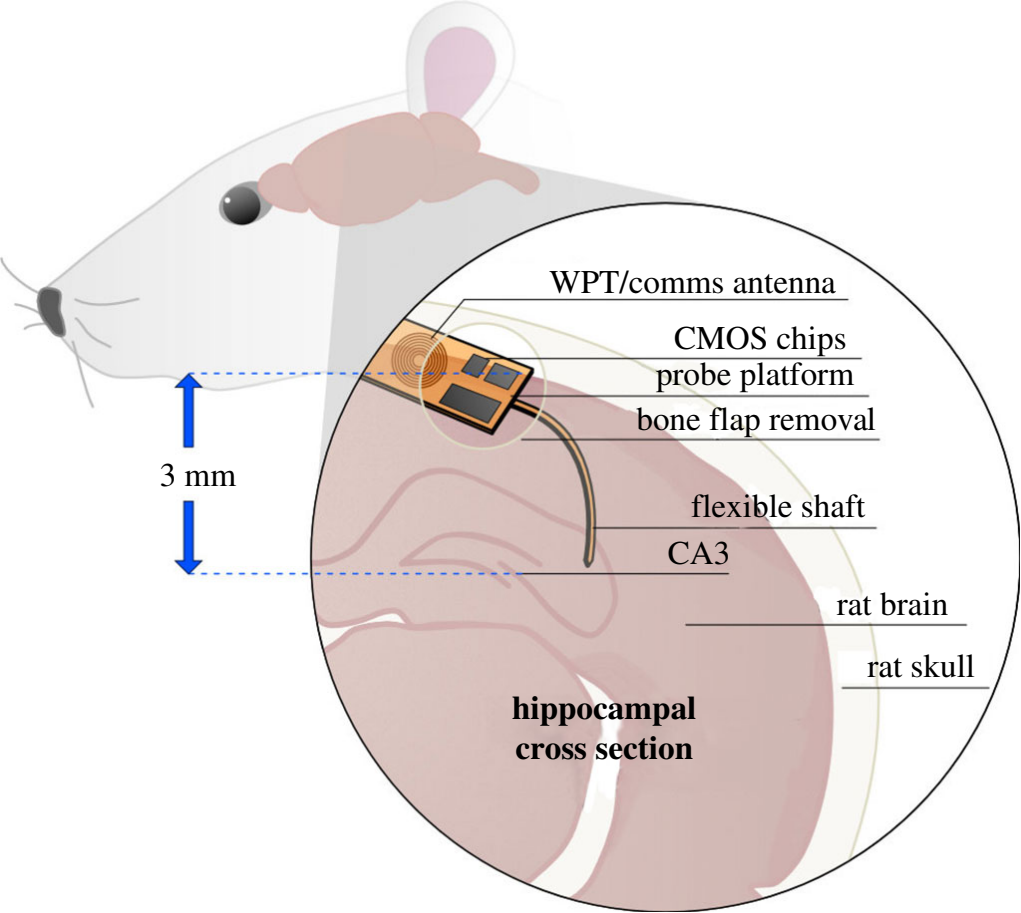
Conceptual diagram of flexible polyimide-based probe, inserted after removal of a rat skull portion. The probe included ultrathin CMOS chips on the base, and bends towards the CA3 region of the hippocampus, approximately 3 mm from the brain surface. Future technologies will incorporate a wireless link on the same substrate as the electrodes. (Online version in colour.)

FLEXIBLE neural probes reduce the likelihood of immune response; however, they introduce increased difficulty in implantation surgery and often require an implantation aid. With the intention of inserting the probe platform under the skin at a right angle to the brain surface, the probe should be easily manipulated during surgery, and robust enough to withstand implantation without buckling, followed by orthogonal bending. Figure 1 illustrates a probe that has been implanted into the CA3 region of the rat hippocampus, with a platform which can be laid flat against the skull by bending the shank (length of polymer which hosts tracks and recording electrodes).

Probes with thin, flexible connections to the outside world are commonly termed ‘floating' probes which exploit microwires or polyimide cables [1–3]. Despite the opportunity to decrease micromotion by foregoing tethering to the skull, the short-lived improvement in recording stability has been dismissed as insufficient justification for the design of floating probes, especially when considering the stringent requirements (shielding and mechanical robustness) for high-frequency communication wires [4]. This work prioritizes a flexible polymer substrate as the first step towards a truly integrated probe for a closed-loop system. In future, miniaturized neural probes which eschew wired connections for the control of stimulation may usurp bulky, battery-powered deep brain stimulation (DBS) systems. While silicon probes boast very high numbers of recording sites [5], it is most common for these probes to stand utterly upright as they protrude from the brain and connect to a headstage outside the body. For neuroscientists to insert a cannula into the rat brain for drug delivery, or to implant a secondary reference electrode, it is ideal for the probe shank to bend at 90°. This could allow for the base of the probe to be tucked underneath the skin of the neck, if there is sufficient shank length.

Countless works by Stieglitz over two decades indicate that despite not being Food and Drug Administration (FDA) approved, polyimides are biocompatible, robust and solid candidates for implantable probe materials [6]. The biocompatibility of polyimides has been rigorously investigated by several research groups [7]. Current experimental results indicate that polyimides are ISO 10993 compatible, although these results must be confirmed with long-term *in vivo* implantation experiments. Polyimides have a decades-long history in neural electronics [6]. In-depth reviews of the literature have previously been carried out by the authors of this work [8,9].

During implantation surgery, there are several opportunities for implant failure. As such, this paper presents a round of simulations to investigate the robustness of the probes to the potential forces incurred during surgery. Further to this, the ideal implantation angle is identified such that the probe creates the lowest possible von Mises stress in the tissue. These results will indicate the suitability of the proposed designs, which will then be produced with nanofabrication techniques, employing a PI-2545 release layer and HD-4110 substrate with Ti/Pt metallization. After release, the probes will undergo mechanical tests which reflect the finite-element simulations.

The structure of this paper is as follows: Section II describes the design methodology and simulation set-up; Section III details the simulation results; Section IV contains the microfabrication process and experimental implantation set-up; Section V is the experimental results; Section VI is the discussion of the findings; and Section VII concludes the paper and provides a future outlook for the discipline.

## 2. Design methodology

### (a) Finite element method

**Figure 2.**
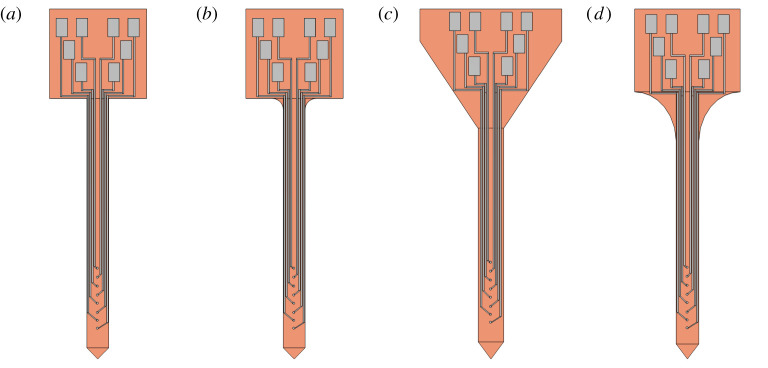
(*a*–*d*) Probes designed in SOLIDWORKS and converted to COMSOL Multiphysics and Tanner L-Edit files. These four designs differ in the shape of the polymer substrate, but share the same electrode pattern. (Online version in colour.)

Four probe shapes, incorporating a platform which measures approximately 1.2 × 1.2 mm, and a 3.35 mm shank (figure 2), were adapted from a previous work [10]. A full description of the preliminary simulations based on the finite-element method using *COMSOL Multiphysics* may be found in our previous work [10], and a similar method has been employed in other publications [11,12] to significant effect. This paper builds on the results of our previous work by incorporating buckling force models, angled implantation simulation and fabrication of the accurate probes with experimental validation. To summarize, beginning with a Solid Mechanics solver module, the four probes were subjected to a Fixed Constraint on the bottom face of the platform, to mimic the contact between the platform and the brain surface. Subsequently, the force exerted on the top face of the polyimide shank was swept between 1 to 100 mN.

Further to this, the buckling force of each probe was calculated. First, a simplified polyimide beam was modelled with varied width and thickness, employing one fixed end (fixed constraint boundary condition) and a total force of 1 mN on the other. This was compared to the numerical result yielded from Euler's critical load equation for a thin beam.

2.1 $$F_{\text{buckling}}=\frac{\pi^{2}EI}{{\left(kL\right)}^{2}}.$$

Conditions for the linear buckling analysis align with a column effective length factor (*k*) of 0.7, applicable to a fixed-hinged geometry. Young's modulus is represented by *E*, *L* is the length and *I* is the moment of inertia of a slender beam. The hinged end was accomplished by placing a prescribed displacement of 0 m along the length direction of the beam. The tip face, which would be in contact with the brain or phantom surface, was also treated as hinged [13]. Linear buckling analysis provided the critical load factor. This value (lambda) was multiplied by the pressure on the tip face in a Surface Integral, yielding the buckling force.

The original design recommendation from [10] was made based on the lowest von Mises stress for a prescribed bending case (90°). Investigation into the implantation protocol for polymer probes suggests that an increased implantation angle and reduced implantation speed will reduce the implantation force [14].

A brain tissue block (density 1040 kg m^−3^) with sides of 5 mm was used for the implantation simulations, recruiting a time-dependent study. A prescribed displacement was placed on the entire probe, equal to the time *t* of the study multiplied by the chosen implantation speed. Since the von Mises stress in the tissue (simulation) is dependent on the implantation distance, rather than the speed, the focus was turned to the implantation angle. The prescribed displacement was repeated, with the angle between the brain surface and the probe varied as previously mentioned.

## 3. Finite-element analysis and simulation results

### (a) Material selection and thickness sweep

Silicon is brittle, suitable for bending when coupled with encapsulation to bridge the gap between rigid areas [15]. Thinning efforts, through etching or grinding, can increase silicon's flexibility. Najafi *et al.* including the creator of the Michigan probe, explored the fracture stiffness of silicon probes thinned to 8 µm, equal to 2 GPa [16]. This value was used as a reference to examine the suitability of silicon as a bendable substrate. Further to the bending that will occur during surgery, any trauma introduced after implantation, such as the rat scratching its headgear, risks snapping the silicon.

**Figure 3.**
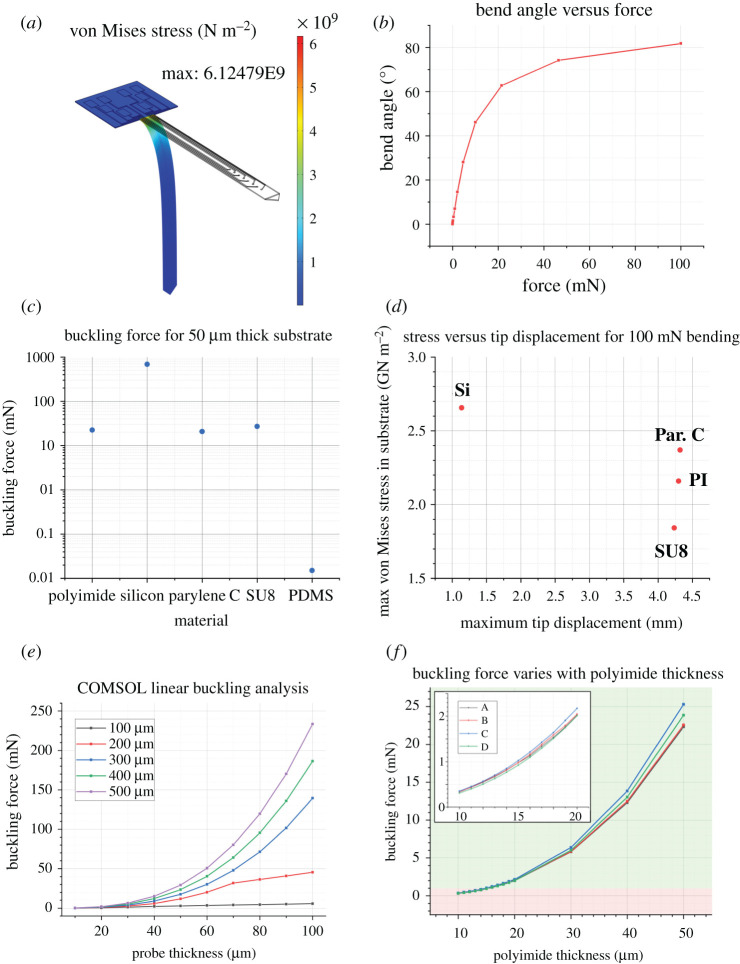
(*a*) Silicon probe with 8 µm thickness, undergoing 100 mN bending force. The maximum von Mises stress occurs at the junction between the platform and the shank. The value is three times higher than the fracture stiffness for silicon as reported by Najafi *et al*. (*b*) The bending angle produced by 100 mN of downward force is 81.8°. (*c*) The buckling force of different substrate materials is compared. (*d*) The bending behaviour of substrate materials is compared. (*e*) Buckling force for a simplified beam with dimensions in the range of hundreds of micrometres. (*f*) The buckling force with respect to probe thickness for each design. The green band denotes a buckling force greater than 1 mN. (Online version in colour.)

In this simulation, the bending case produces a maximum von Mises stress of 6.12 × 10^9^ N m^−2^ (equivalent to 6.12 GPa), as illustrated in figure 3*a*. As such, this probe does not meet the criteria for orthogonal bending (the platform is flat to the brain surface and the shank implanted into the deep brain). Even when the probe tip is 81.8° to the platform (figure 3*b*), the von Mises stress suggests the probe would fracture. For this design, silicon is not an appropriate substrate.

Four common neural probe substrate polymers were also compared, and their most essential characteristics are summarized in table 1: polyimide, parylene C, SU8 and PDMS. The buckling force of each was evaluated for a 50 µm thick substrate, though the bending behaviour at 100 mN downward force could only be evaluated for polyimide, silicon, parylene C and SU8. PDMS achieves orthogonal bending under forces as low as 0.1 mN, not considered in figure 3*d*. Due to Young's modulus similarity, polyimide, parylene C and SU8 show no significant difference in either buckling force or maximum tip displacement under 100 mN bending force.

**Table 1.** Mechanical characteristics of popular polymer probe substrate materials.

material	Young's modulus (GPa)	Poisson ratio	density kgm^−3^
polyimide	2.5	0.34	1300
PDMS	0.36–0.87	0.5	970
parylene-C	2.8	0.4	1652
SU8	4.02	0.22	1200
silicon	170	0.28	2329

As such, due to existing expertise in the James Watt Nanofabrication Centre focused on the use of polyimide, it was selected as the candidate material.

### (b) Buckling force

COMSOL results for the buckling force of simplified beams aligned well with results from Euler's beam equation (used to calculate the numerical result in table 2). The most relevant case in this instance is the 50 µm thickness, 300 µm width beam, which when fabricated using polyimide, will have a buckling force of 17.5 mN. It is essential to determine the buckling force of the actual probe geometries, not only a simple column. These results are displayed in table 2.

**Table 2.** Buckling force for four designs of neural probe.

probe design	*F*_buckling_ (mN)	cross-sectional area (µm^2^)	shank length (µm)	numerical *F*_buckling_ (mN)
A	22.35	14 600	3350	15.31
B	22.52	14 600	3200	16.78
C	25.30	17 136	2867.57	33.78
D	23.85	15 018	2700	25.65

The shape of the probe shanks under a buckling load indicates good agreement with the choice of *k* value (used in the numerical calculations), aligning with the theoretical buckling shape for a clamped-hinged case. Disparate platform shapes dictate the maximum shank length, most evident in design *C*.

### (c) Bending conditions

**Figure 4.**
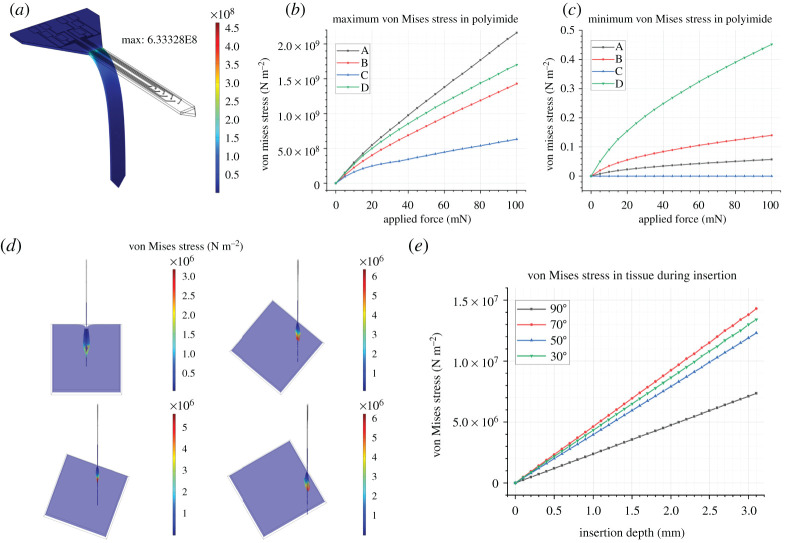
(*a*) Illustration of the von Mises stress in a probe bent at a large angle, around 80°. (*b*) Maximum von Mises stress for each design *a*–*d* in the polymer. (*c*) Minimum von Mises stress for each design *a*–*d*, in the polymer. (*d*) Illustration of the brain block at *x* = 1100 µm, also known as the point of probe implantation. Classic dimpling can be seen most clearly for the perpendicular implantation case, though the von Mises stress in the tissue is lowest. (*e*) The von Mises stress in the tissue is significantly lower for the perpendicular implantation case compared to the 70°, 50° and 30° instances. (Online version in colour.)

Figure 4*a* illustrates that when a 100 mN force is incident on the probe shaft, each of the designs bends to a high angle (80° ± 1°). Therefore, any of the designs are suitable candidates for the perpendicular bending case. In figure 4*b*, the maximum von Mises stress occurs in the topmost part of the shank, where the radius of curvature is greatest.

Each different probe type (A-D) experiences the bending force in a different manner: this is most obvious in the case of figure 4*c*, in which the platform section of probe C is not displaced at all, and as such, experiences zero von Mises stress. Design C has the lowest von Mises stress as it undergoes bending.

### (d) Implantation

While the engineering focus of this work is mainly on the design of the neural probes, simulation can inform surgical techniques and reduce harmful stress in the brain tissue during implantation, by highlighting the ideal angle for implantation. The COMSOL implantation model does not reflect the piercing behaviour of a neural probe during implantation into a brain slice or brain phantom. In this instance, when the probe tip is at a right angle to the brain surface, the von Mises stress in the tissue is significantly lower than any of the other implantation regimes.

Typically, implantation experiments involve varying the implantation speed of the probe: in this case, at the maximum implantation depth, the von Mises stress in the tissue was almost indistinguishable between implantation speed simulations. As such, the implantation speed was kept constant at 1 mm s^−1^. Further investigation into the impact of implantation angle was carried out after the fabrication of the probes was completed and could be manually inserted into an agarose brain phantom.

**Figure 5.**
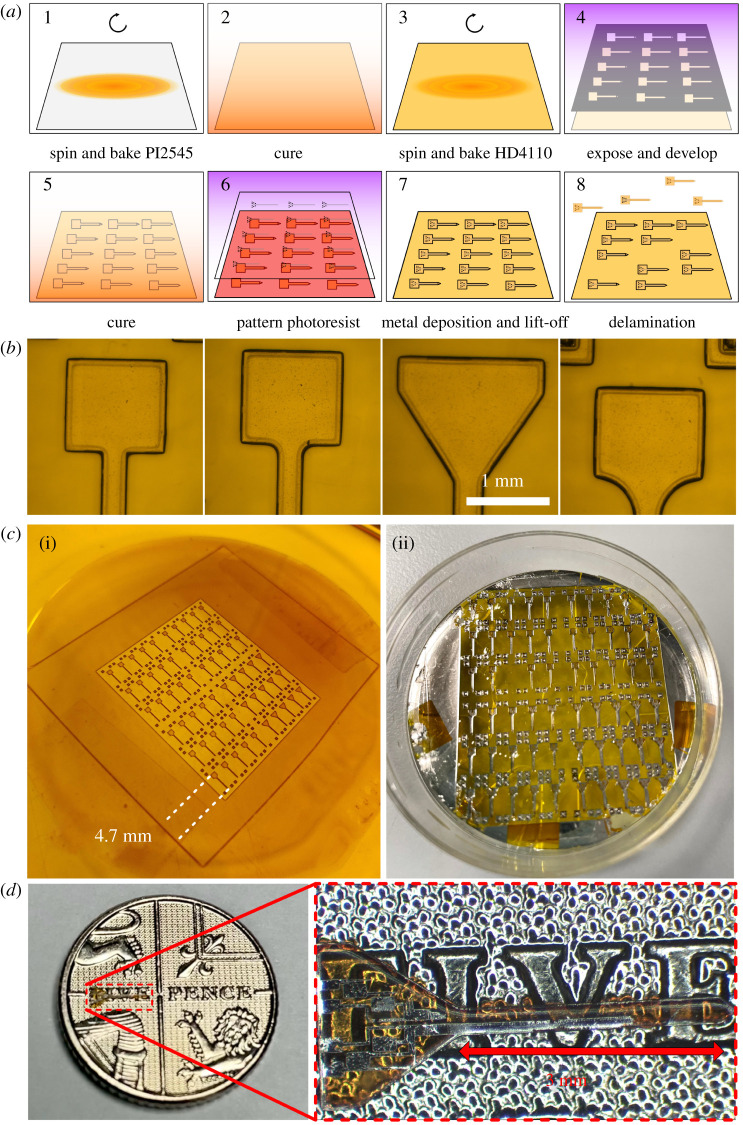
(*a*) Fabrication steps for miniaturized polyimide probes, beginning with a clean glass substrate, stepping through the spinning and patterning process, and ending with the delamination process in water after which point the probes may be removed from the glass. (*b*) Developed polyimide for four designs. (*c*) (i) Cured sample from above, indicating the outline of the acetate mask used in research and development. (ii) After metal deposition on a 2-inch wafer, the glass is soaked overnight in reverse osmosis water to encourage delamination. In this image, a single probe has been peeled off, with the others to follow. (*d*) Delaminated probe on top of a £0.05 coin (18 mm in diameter) for scale illustration only. The probe is transparent and allows the text to be viewed through it. (Online version in colour.)

Most importantly, the 90° implantation case has much lower tissue stress than the other techniques. Figure 4*d* shows a maximum von Mises stress (for the *x* = 1100 µm cross-section) in the tissue of 3.20 MPa for the 90° case, and 5.68 MPa for the 70° case. Figure 5*e* illustrates that the difference in maximum von Mises stress across the whole tissue block is even more pronounced, with the 90° case clearly the most appropriate.

## 4. Microfabrication and implantation experiment

### (a) Flexible probe fabrication

The critical steps of the fabrication protocol are summarized in figure 5*a*. Borosilicate glass was chosen as the rigid substrate, which can be reused after removing the probes and release layer. Two inch diameter wafers (*PI-KEM*) were cleaned in 5-min rounds of sonication in Opticlear, acetone and methanol before being rinsed for five minutes in flowing reverse osmosis (RO) water and dried with N_2_ gas.

PI-2545 (*HD Microsystems*) was spin-coated onto the surface of the glass. This was cured at a range of temperatures up to 300°C in an oven which had been flushed with nitrogen gas, in line with the manufacturer's instructions. A single layer of the photo-definable polyimide, HD-4110 (*HD Microsystems*), was spin-coated on top. The film thickness was measured to be 57.7 µm using a Dektak XT profiler (Bruker), close to the intended thickness of 50 µm.

An acetate photomask (*Microlithography Services Ltd*) was used for the prototype. Exposure was carried out with a SUSS MA6 mask aligner. The HD-4110 was then developed in cyclopentanone and rinsed in propylene glycol methyl ether acetate (PGMEA). The polyimide was fully cured as before. The patterns produced in the HD-4110 are illustrated in figure 5*b*,*c*. Using an electron-beam evaporator (Plassys MEB550S), 10 nm Ti and 90 nm Pt were deposited and patterned with a standard lift-off process, employing LOR3A and S1818 resists.

The PI-2545 film and HD-4110 probes were released from the borosilicate carrier substrate by submerging the polyimide in RO water overnight, after which point the release layer and probes could be removed with tweezers. PI-2545 acts as a very successful release layer, mainly because of its flexibility, making it simple to manipulate the film and peel the probes away with no damage. Similar approaches use PI-2611, another non-photo-definable polyimide [17]. A released probe is shown in figure 5*d* for scale.

### (b) Brain phantom preparation and implantation

**Figure 6.**
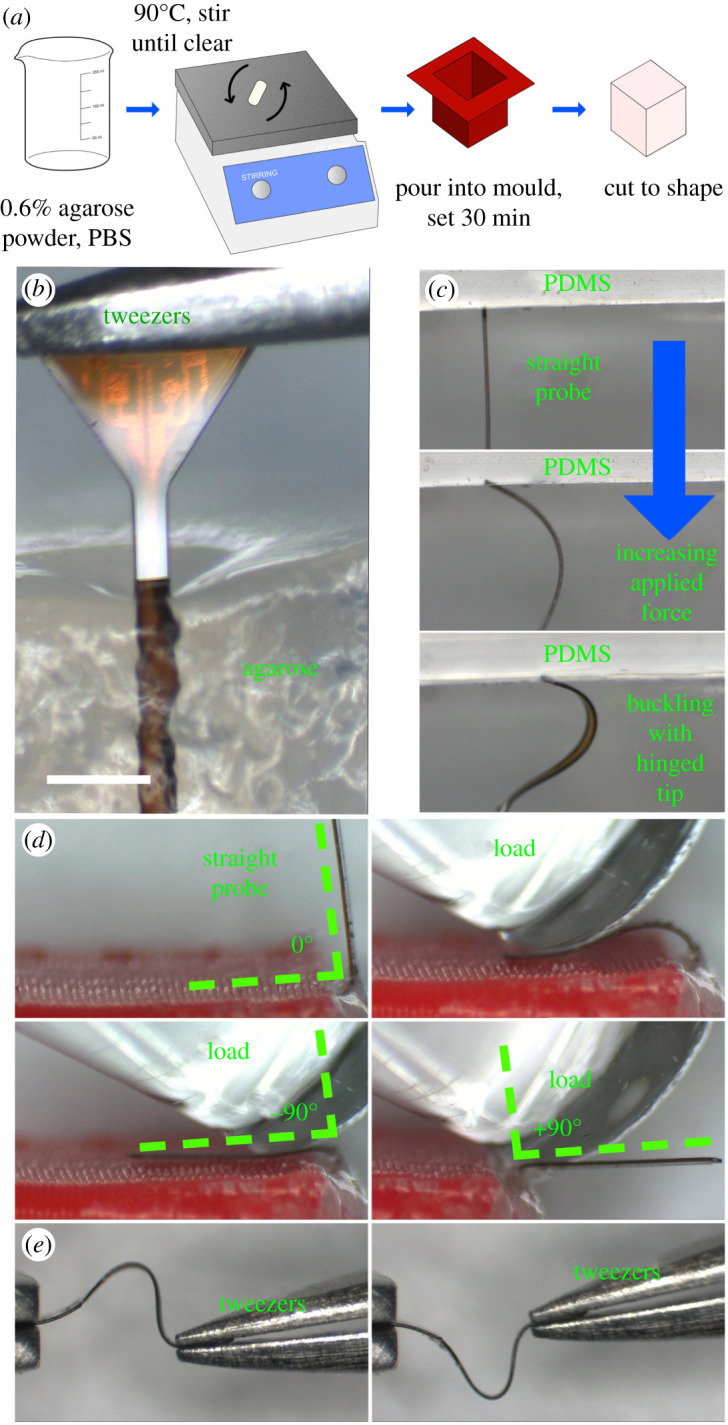
(*a*) Fabrication steps for an agarose brain phantom. (*b*) Agarose surface dimpling during successful implantation of a flexible probe (*c*) The probe buckles as it is pushed against the PDMS surface. (*d*) Orthogonal bending of the probe stem as required to insert the platform under the skin at a right angle to the stem. (*e*) Uniaxial bending of the polymer probe using two tweezers. (Online version in colour.)

Insertion into lamb brain was compared to 0.6% wt agarose gel. Gels prepared to concentrations of 0.6% have Young's modulus of around 10 kPa, similar to the brain tissue [18]. Direct comparisons between agarose, silicone and rat brain concluded that the substitution of cerebrospinal fluid for deionized water (in the rat brain and agarose phantom, respectively) reduces the implantation force into the agarose, quickly dissolving any stiffener coating [18]. Despite this, it is widely accepted as a brain phantom material [19–22]. Agarose phantoms were prepared in a cuboid mould (figure 6) with a side length of 35 mm using 0.6% *Sigma Aldrich* Agarose powder and phosphate-buffered saline (*Fisher Scientific*). The solution was stirred on a magnetic hotplate for at least 10 min, raised to a temperature of around 90°C. When the agarose powder was completely dissolved, the beaker was removed from the hotplate and poured into the mould. Within 30 min, the phantom was set.

As per the buckling force and implantation angle simulations carried out earlier in this work, an Instron 5966 Universal Testing machine was used to control the speed of insertion in compression experiments. A Honeywell Model 31 50 g load cell was used for force sensing on the scale of mN. First, the insertion force was investigated. Buckling force tests were carried out with new probes, in case of any mechanical damage from the clamping process. The probe tip was pressed directly against the top three-dimensional printed plate mounted on the load cell until buckling occurred at a constant speed of 1 mm s^−1^. The buckling force was measured for each sample.

An agarose brain phantom was prepared from a mixture of phosphate buffered saline and 0.6% wt agarose powder (*Sigma Aldrich*). The solution was poured into 35 mm silicone moulds and allowed to cool at room temperature. An agarose phantom was then sliced to correspond to each of the target angles (30°, 50°, 70° and unsliced at 90°). From here, the probe was lowered at a rate of 1 mm s^−1^. The insertion success rate was noted for each sample. Similarly, lamb brain samples were mounted on a three-dimensional printed plinth, and the insertion success rate was compared to the 90° agarose block.

### (c) Three-point bending tests

Using a Nordson DAGE 4000Plus Bond tester, three-point bending tests were carried out on 20 probes selected at random, five from each design. The Push-Pull cartridge and three-point bending test head allow for a force range up to 500 g or 5 N, with a 1 mm wide blade to impart the force in the centre of the probe. Due to its small dimensions, care was taken to position the mounts for the probe such that it was equidistant, and the blade came down in the middle of the total length. The 100 g force range was selected in the software set-up, the lowest available for that cartridge.

From the Micro Bending template, the following parameters were adjusted: sample width, sample thickness, loading rate (corresponding to a test speed of 50 µm s^−1^, test load (20 mN), and maximum pull distance (1 mm)). Before each test began, the *z*-height of the test blade was lowered to the same value such that the blade would touch down on the probe surface at the same point. However, it is not guaranteed that the probes are perfectly straight and flat, which means that the useful force/distance gradient begins at a different point in each experiment.

The results from each of the probe styles were compared to ascertain the effect of the geometry on the force/distance curve. Similar to the buckling force equation, the probe may be modelled as a rectangular beam. The gradient of the force/distance graph is proportional to Young's modulus (E) as per this equation

4.1 $$\omega_{0}=\frac{PL^{3}}{48EI}.$$

The deflection $\omega_{0}$, equivalent to the distance axis on the results graph, is equal to the applied force *P*, with *L* as the length of the beam and *I* the moment of inertia as before. Ergo, Young's modulus is proportional to the applied force divided by the distance travelled.

### (d) MRI compatibility tests

A 12 ml syringe was filled with six agarose layers, prepared in the same manner as the phantoms. The first layer was formed by pulling 2 ml of agarose liquid into a syringe and placing it nozzle-up in a degasifier for a few moments. This caused violent bubbling after only a few seconds and the degasifying process terminated. A probe was placed between each layer in a different orientation and the lower layer was allowed to harden for 10 min before the next was placed. Each subsequent layer was poured on, after degasifying the liquid in a beaker for a few moments. This removed the small bubbles which threaten to create artefacts during imaging.

Scans were performed in a Bruker PharmaScan 7 T MRI machine in the Glasgow Experimental MRI Centre (GEMRIC) [23]. Short initial scans were performed in a 35 mm coil, then the phantom and probes were scanned at a higher resolution overnight.

## 5. Results

### (a) Buckling tests

Two 2-inch glass wafers, processed described in the previous section, yielded at least 25 of each probe design A-D. A PDMS phantom was used as a comparison to the agarose implantation. While soft silicones such as Ecoflex-0010 [18] may be used as an alternative to brain tissue for mechanical tests, Sylgard 184 PDMS has Young's modulus of close to 1 MPa [24], while Young's modulus of brain tissue is around 1 kPa [25]. As such, it was expected that implantation would fail: more importantly, the probe exhibited the expected buckling shape and behaviour with a hinged contact point between the probe tip and the PDMS (figure 6*d*).

**Figure 7.**
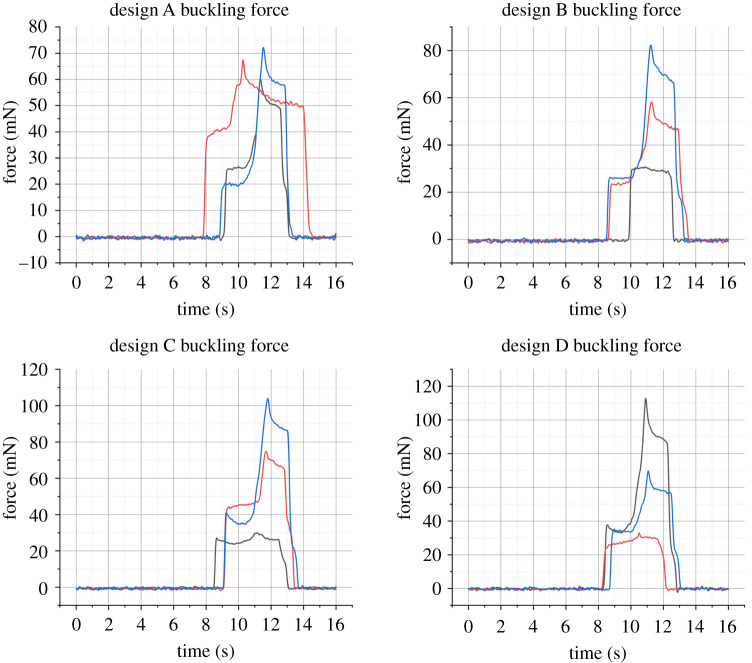
Buckling force measured with Honeywell 50 g load cell. Buckling occurs at the first peak of the trace, with values ranging between 20.42 and 41.16 mN. (Online version in colour.)

The results of the buckling tests may be viewed in figure 7. These graphs were smoothed in Matlab using a Gaussian filter with a window size of 50. Force increases to a point (between 20.42 and 41.16 mN) before buckling occurs. Then the measured force rises again until the loading is completed. This is in good agreement with the simulations, however the range of buckling forces measured between probes of the same design speaks more to how the probes are mounted than any variation between designs. One future avenue of investigation to guarantee the mounting is the same between probes would be to three-dimensionally print a custom holder.

### (b) Insertion tests

**Figure 8.**
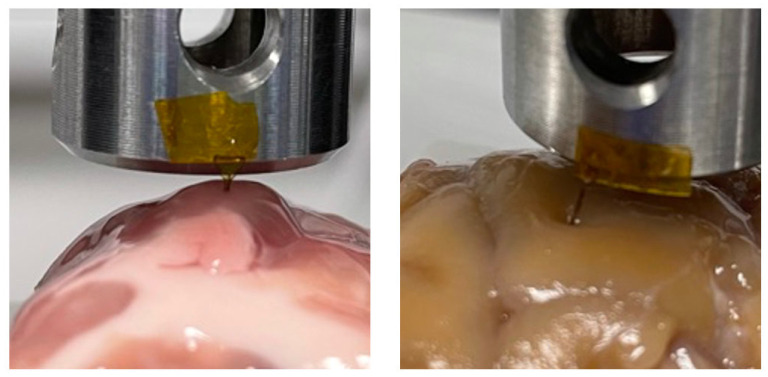
Insertion using Instron 5966 travelling at 1 mm s^−1^. Insertion was successful in deeper regions of the brain tissue, and unsuccessful when attempted through the pia mater. (Online version in colour.)

First, insertion was performed into freshly prepared agarose, covered with Parafilm when not in use. The success rate for each probe design, as well as the success rate at various angles, was investigated and found to be 100% at a rate of 1 mm s^−1^. However, agarose does not properly model the effect of the pia mater or varying Young's modulus depending on the region of the brain. Insertion failed when the probe was pressed directly against the pia mater, but was 100% successful in deeper regions of the brain which were not coated in pia mater. Images of the lamb brain insertion may be viewed in figure 8.

### (c) Three-point bend tests

**Figure 9.**
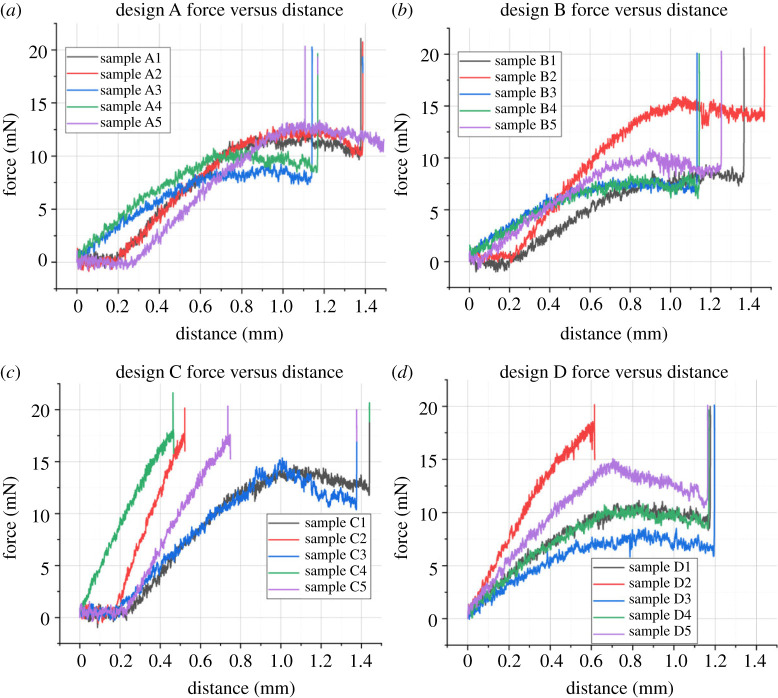
Force versus distance results for four designs (*a*–*d*) of neural probes, collected during three-point bending tests using a DAGE 4000Plus Bond Tester with a Push-Pull 500 g cartridge and three-point bend test head. The maximum force employed was 20 mN, and the probes could be pushed down by 1 mm comfortably without undergoing permanent bending. All probes were processed with the same test settings, however, the tester halted downward motion at a different point for each probe. The force exerted by the stationary test head was ramped to 20 mN in each case. (Online version in colour.)

The probe's flexibility is shown in figure 6*e*, which also provides a sense of scale when comparing the probe to the metal tweezer tip. This is further explored in the three-point bending test, as the middle of the probe is deflected by 1 mm without any permanent damage. The total length of the probes is less than 4 mm. While optimizing the parameters of the three-point bend experiment, the probe withstood forces up to 70 mN with no adverse effects. Figure 9 shows the steady rise in applied force before the maximum load of 20 mN was applied. The most critical part of these graphs is the gradient between the beginning of loading and the maximum force spike. Most notably, designs C and D show higher gradients than designs A and B. The three-point bending test illustrates the subtle differences the probe design (specifically the platform-shank junction) has on the loading conditions for bending after implantation. Visual inspection of the probes after bending showed no permanent deflection or damage.

### (d) MRI scan

**Figure 10.**
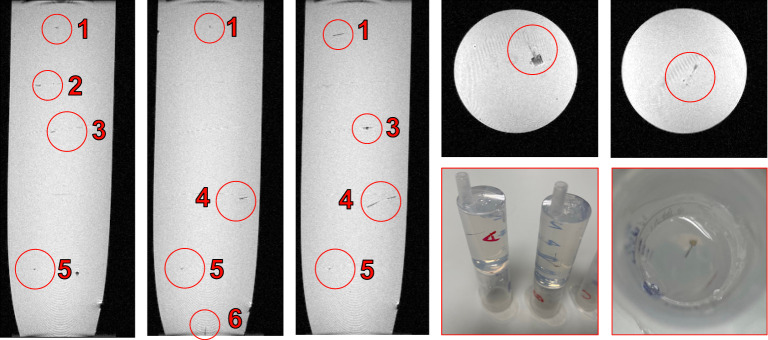
MRI scan showing seven layers of agarose phantom inside a 12 ml syringe, containing six probes in different orientations. No artefacts are witnessed around the probes, nor did the probes move under a magnetic field. (Online version in colour.)

Results of an overnight MRI scan are illustrated in figure 9. The nature of the agarose gel meant that control over the orientation of the probes was limited after the gel was poured. Of the six probes in the 12 ml gel, five were laid flat on the gel surface (on the *x-y* plane in figure 10), and one was implanted vertically. The probes showed no significant artefacts regardless of orientation. Any artefacts witnessed in the gel may be attributed to small air bubbles, though the best attempts were made to create a gel which was as homogeneous as possible. Despite this, the layers may still be clearly seen. The slight differences in probe geometry show no impact on the number of artefacts in the scan.

## 6. Discussion and recommendation

The most crucial mechanical characteristic of the neural probes is that their buckling forces, predicted to range between 22.35 and 25.30 mN for 57.7 µm thick polyimide, exceed the implantation force for implantation into an agarose phantom. Investigating the impact of thickness on the buckling force, the minimum acceptable thickness of design *C* is 15 µm, which has a 1.03 mN buckling force.

For bending forces up to 100 mN, the maximum angle produced is 80°, with a maximum von Mises stress in the polyimide of over 500 MPa. However, the flexible probes show no evidence of fracture. To further reduce the likelihood of significant immune response, the width and thickness should be reduced as far as possible, decreasing the track width and spacing while maintaining the appropriate length to reach the CA3 region. The gradient of the force/distance graph produced by the three-point bend test for probe C4 is equal to 32.38, while probe A1 has a gradient of only 17.725. As the material composition of the probes is almost identical, the discrepancy may be attributed to the changes in the probe geometry.

The 50 µm polyimide probe selected in [10], while similar in shape to design *D* from this work, has been improved by adding six further electrodes with corresponding tracks and pads. Once the probe platform was enlarged to allow space for eight electrodes, design *C* emerged as the front-runner for both buckling load and maximum von Mises stress.

Current literature is limited when it comes to simulating the implantation behaviour of a neural probe into brain tissue. Similar topics which involve the same physics, such as the implantation of microneedles [26,27], explore the buckling force and skin dimpling which occurs during microneedle implantation. While the neural probe in these simulations undergoes a prescribed displacement and moves into the brain block, it does not achieve the same slicing effect necessary for implantation into the brain, after which point the implantation forces are significantly reduced. Previously reported neural probe implantation simulations using *COMSOL Multiphysics* also did not capture the piercing behaviour [28]. Further limitations of the simulations include the fact that despite employing a viscoelastic rat brain model, implantation speed does not noticeably impact the von Mises stress which results from the implantation.

A crucial finding of this work is that the von Mises stress in the tissue is significantly lower for the 90° case. The correlation between von Mises stress and strain indicates that strain in the brain tissue is decreased by the 90° implantation method, which is encouraging for the long-term health of the tissue surrounding the implant. For example, the ‘kill zone' described by Hamzavi *et al.* is defined as regions with more than 5% strain [29]. Stress at the implantation angle *θ* is compared numerically to the 90° case as per equations ((6.1)–(6.8)) adapted from Halabian *et al.* [30].

6.1 $$\begin{array}{rl} \sigma_{z90^{\circ }} & \;=\frac{F}{A}, \end{array}$$

6.2 $$\begin{array}{rl} \sigma_{z\theta } & \;=\frac{P}{A}\pm \frac{M_{y}c}{I_{y}}=\frac{P}{A}\pm \frac{Vzc}{\frac{1}{12}ab^{3}}=\frac{P}{A}\pm \frac{Vz\frac{h}{2}}{\frac{1}{12}Ab^{2}}, \end{array}$$

6.3 $$\begin{array}{rl} \sigma_{z\theta } & \;=\frac{\text{Fcos}\theta }{A}\pm \frac{F\sin\theta z\frac{h}{2}}{\frac{1}{12}Ab^{2}}=\left(\cos\theta \pm \frac{6\sin\theta hz}{b^{2}}\right)\sigma_{z90^{\circ }}, \end{array}$$

6.4 $$\begin{array}{rl} \sigma^{\prime } & \;=\frac{1}{\sqrt{2}}{\left[{\left(\sigma_{x}-\sigma_{y}\right)}^{2}+{\left(\sigma_{y}-\sigma_{z}\right)}^{2}+{\left(\sigma_{z}-\sigma_{x}\right)}^{2}+6(\tau_{xy}^{2}+\tau_{yz}^{2}+\tau_{zx}^{2})\right]}^{\frac{1}{2}}, \end{array}$$

6.5 $$\begin{array}{rl} \tau_{xy} & \;=\frac{V}{A}=\frac{F\sin\theta }{A}=\sigma_{z90^{\circ }}\sin\theta , \end{array}$$

6.6 $$\begin{array}{rl} \sigma_{\theta }^{\prime } & \;=\frac{1}{\sqrt{2}}{\left[{\left(0-0\right)}^{2}+{\left(0-\sigma_{z\theta }\right)}^{2}+{\left(\sigma_{z\theta }-0\right)}^{2}+6(\tau_{xy}^{2}+0+0)\right]}^{\frac{1}{2}}, \end{array}$$

6.7 $$\begin{array}{rl} \sigma_{\theta }^{\prime } & \;=\frac{1}{\sqrt{2}}{\left[2{\sigma_{z\theta }}^{2}+6{\left(\sigma_{z90^{\circ }}\sin\theta \right)}^{2}\right]}^{1/2} \end{array}$$

6.8 $$\begin{array}{rl} \text{and }\;\sigma_{\theta }^{\prime } & \;=\frac{1}{\sqrt{2}}{\left[2{\left(\cos\theta \pm \frac{6\sin\theta hz}{b^{2}}\right)}^{2}+6{\left(\sin\theta \right)}^{2}\right]}^{1/2}\sigma_{z90^{\circ }}. \end{array}$$

$\sigma_{z90^{\circ }}$ is the normal *z*-direction stress for the 90° case, which is equal to the perpendicular force (*F*) over the area of the tissue (*A*). In turn, the area is equal to the length (*a*) multiplied by the width (*b*). The height (*h*) of the tissue is the maximum possible value for the implantation depth (*z*) of the probe. This value, *z*, changes according to the implantation angle. $M_{y}$ is the bending moment, taken from the flexure formula, while the distance from the neutral axis is depicted by *c*, and the surface moment of inertia by $I_{y}$. Finally, the von Mises stress, $\sigma^{\prime }$ incorporates the shear stress, *t*.

**Figure 11.**
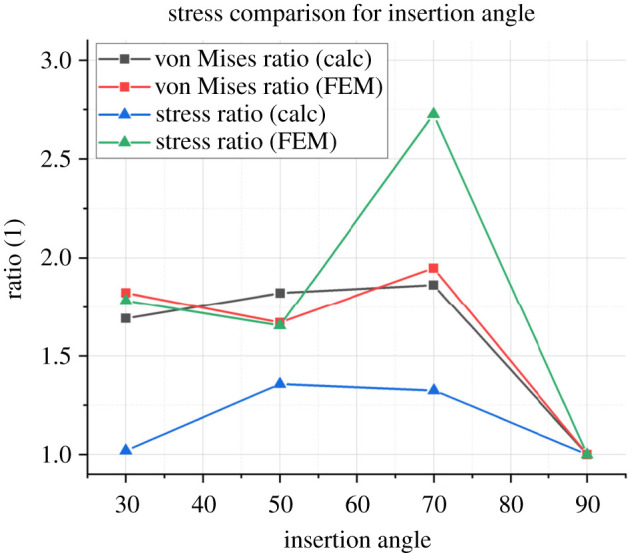
The ratio illustrated on the *y*-axis compares the values of each stress at different angles to the original 90° case, e.g. the theoretical von Mises stress for the 70° case divided by the theoretical von Mises stress for the 90° case. The stress ratio is calculated using equation (6.3) while the von Mises ratio is calculated using equation (6.8). This follows the procedure set out by Halabian *et al*. (Online version in colour.)

The results of these equations (stress in the *z*-direction and von Mises stress) were compared to the corresponding finite-element values for the stress tensor in the *z*-direction and the von Mises stress in the tissue. A probe was simulated with an implantation speed of 1 mm s^−1^ at the 1 s point, after which time the implantation depth would be equal to sin⁡θ in millimeters using basic trigonometry. Figure 11 illustrates the ratio between the stress values for implantation at θ versus implantation at 90°. In both the calculated and FEM results, the maximum von Mises stress occurs at 70°. Both the von Mises stress and stress tensor are at a minimum for 50° implantation, which does not agree with the numerical results. The probe tip position may account for this error, which cannot be precisely placed against the tissue surface at lower implantation angles.

A simple fabrication process is made possible by combining a polyimide release layer and a photo-definable polyimide substrate. PI-2545 is a standard sacrificial layer, typically removed with dry etching. In this work, the poor adhesion of PI-2545 to glass and HD-4110 is exploited: not only does this remove the need for a time-consuming dry etch step, but it produces free probes made from photo-definable HD-4110. This combination of polyimide significantly reduces the complexity of the fabrication process. Unlike previous approaches using a PI-2611 release layer, there is no need to cut through the film before removing it from the rigid substrate [17].

While the physical probe produced using this protocol is thicker than many of the examples cited in table 3, the lowest buckling force recorded (20.42 mN) is 1.27 times greater than the next highest value [19] which is made from silicon, at 16 mN for a 10 µm thick probe. As such, this creates significant scope to miniaturize the design further. This work has comparable dimensions to [33], which uses a microfluidic channel to ensure adequate buckling force for implantation.

**Table 3.** Performance summary and comparison table of this work with recently published neural probes.

material	dimensions	thickness	implantation media	implantation aid	buckling force	successful implantation	year	ref.
polyimide	342 µm × 3.35 mm	57.7 µm	agarose gel	none	20.42–41.16 mN	yes	2022	this work
polyimide	250 µm × 10 mm	9.5 µm	gelatin hydrogel	steel needle	—	yes	2020	[31]
parylene	110 µm × 1.2 mm	20 µm	flat agarose mould	PEG mould	0.52 mN	no	2020	[20]
silicon	—	10 µm	agarose model with and without pia model	none	16 mN	yes	2020	[19]
cyclic olefin polymer	650 µm width	50 µm	—	none	8 mN yield stress	—	2019	
parylene and SU8	90 µm width	20 µm	agarose gel	PEG mould	1.9 mN uncoated, 9.82 mN coated (calculation)	yes	2019	[32]
PDMS/PI	200 µm width	76 µm	agarose gel	microfluidic channel	0.25–1.25 mN	yes	2019	[33]
silicon	70 µm × 5 mm	40 µm	rat brain	none	—	yes	2018	[34]
polyethylene, shape memory polymer	6 mm × 13.5 mm (PE dummy)	75 µm	agar gel and rat motor cortex	PMMA slotted guide	3.8-fold increase with guide	yes	2018	[35]

While 0.6% wt agarose gel is typically used as a brain phantom, it is limited in that it does not reflect the pia mater layer. In this work, the probes were 100% successful when inserted into agarose, but could not pierce the pia mater. This indicates that at the very least, an incision would be required into the pia to allow for implantation.

The findings of this work stand apart from the current literature in neural probe design in that the focus of the finite-element simulations is not the impact of micromotion, as with several of the papers summarized in table 4. In this work, three rounds of simulations informed the choice of probe geometry and implantation angle. It is clear from the state of the art that further investigation is required to assess the optimal implantation scheme, since each paper cited in table 4 assumes the probe is already inserted into the tissue. While small longitudinal displacements are investigated, insertion simulations are limited for neural probes specifically.

**Table 4.** Recent works employing finite-element method simulation to inform probe design.

topic	design impact	year	ref.
buckling, bending and implantation	wide shank with ‘strain relief’ platform shape, PI as candidate material, orthogonal insertion reduces stress	2022	this work
buckling and micromotion	PDMS/PI probes have smaller displacement than PI probes under micromotion	2019	[33]
transient micromotion	reduce Young's modulus of material to 200 kPa	2016	[36]
micromotion	fillet radius of 20 µm and wedge angle of 70° reduces tissue damage	2016	[37]
transient pulses of micromotion	stiffness of probe should be less than three orders of magnitude larger than the brain stiffness	2014	[38]
‘large’ 25 µm micromotion	polymers generate a smaller ‘kill zone’	2013	[29]
longitudinal and transverse micromotion	von Mises strain field increases with friction coefficient	2011	[39]
*z*-displacement in implanted probe to investigate interfacial forces and tethering	reducing tethering, minimizing the probe stiffness and increased interfacial adhesion may reduce the tissue strain	2005	[40]

## 7. Conclusion and future work

In this paper, four different probe structures were fabricated and studied to determine the most robust probe base shape, implantation angle with the least tissue stress, and optimal material for a neural probe with careful finite-element analyses using *COMSOL Multiphysics*.

First, the proposed eight-electrode designs were verified in mechanical finite-element simulations: the buckling force of the optimum design (C, triangular base) was 23.65 mN, significantly higher than the 1 mN required buckling force for implantation into rat brain. This meant that the probes could be easily inserted into a brain phantom with no external aid. Low bending forces around 100 mN are required to produce a ±90° bend in the polyimide probe, after which point the probe recovers and regains its original shape without fracturing. From the simulation, the implantation case with the lowest tissue stress is at a right angle to the brain. The probes were easily implanted in an agarose phantom but failed to pierce the pia mater of the lamb brain sample. In future, the fabricated polyimide-based probes require two avenues of further investigation.

### (a) MRI- and bio-compatibility

This work is part of a larger body of work focused on developing flexible, miniaturized neural probes to treat epilepsy. Incorporating state-of-the-art materials, with encouraging results in biocompatibility tests, modern neural probes seek to strike the perfect balance between flexibility and simple implantation surgery. By tuning the buckling force of a polymer probe, there is an opportunity to eradicate bulky stiffeners, increasing tissue damage. Thin electrode layers, recruiting MRI-compatible materials, ensure that the implantation of flexible neural probes does not prohibit patients from undergoing MRI in the future. In this work, the polyimide substrate is the only polymer layer considered: in reality, *in vivo* tests and electrochemistry measurements would require an encapsulation layer, exposing only the electrodes and pads. Examples include parylene-C and silicone. Combining polyimide with an encapsulant layer which encourages cell adhesion and shows no toxicity to tissue is paramount for the final probe design.

## Data Availability

McGlynn E, Walton F, Das R, Heidari H. 2021 Instron 5966 Buckling data from Oscilloscope. (https://doi.org/10.6084/m9.figshare.17306762.v1). McGlynn E, Walton F, Das R, Heidari H. 2021 DAGE 4000Plus Three-point bend test data. (https://doi.org/10.6084/m9.figshare.17306786.v1).
